# Evaluating the Microstructures and Mechanical Properties of Dissimilar Metal Joints Between a New Cast Superalloy K4750 and Hastelloy X Alloy by Using Different Filler Materials

**DOI:** 10.3390/ma11102065

**Published:** 2018-10-22

**Authors:** Jilin Xie, Yingche Ma, Meiqiong Ou, Weiwei Xing, Long Zhang, Kui Liu

**Affiliations:** 1 Institute of Metal Research, Chinese Academy of Sciences, No. 72 Wenhua Road, Shenyang 110016, China; jlxie16b@imr.ac.cn (J.X.); mqou@imr.ac.cn (M.O.); wwxing@imr.ac.cn (W.X.); longzhang@imr.ac.cn (L.Z.); 2 School of Materials Science and Engineering, University of Science and Technology of China, No. 96 Jinzhai Road, Hefei 230026, China

**Keywords:** dissimilar metals, new cast superalloy, Hastelloy X, microstructures, mechanical properties

## Abstract

Two kinds of filler materials were used to join dissimilar alloys between a new cast superalloy K4750 and Hastelloy X by tungsten gas arc welding (GTAW). The segregation behavior, interfacial microstructure and mechanical properties of the dissimilar joints were evaluated. The results show that both filler materials can be used to obtain sound dissimilar joints successfully. Microstructural observation show that no obvious cracking is observed in the joints achieved by both filler materials. The segregation extent of various elements in Hastelloy X weld metal is more severe than that in the K4750 weld metal. No unmixed zones were observed at the interfaces. Transition areas with the chemical compositions various between the K4750 alloy and the Hastelloy X alloy were found at the joint interfaces. The maximum width of the transition area between the K4750 weld metal and Hastelloy X base metal is smaller than that between the Hastelloy X weld metal and K4750 base metal. The ultimate tensile strength and yield strength of the joints with Hastelloy X filler material are slightly higher than those with K4750 filler material, however, the K4750 filler material results in a higher total elongation and fusion zone microhardness than those with Hastelloy X filler material. Both dissimilar joints fractured with a ductile feature which exhibits tearing edges and dimples. Hastelloy X filler material is suggested to be more suitable for joining of K4750 superalloy and Hastelloy X dissimilar metals in terms of obtaining superior comprehensive mechanical properties.

## 1. Introduction

Recently, a new cast and precipitation hardened superalloy named K4750 was developed in China. It shows excellent comprehensive properties including great mechanical properties, hot-corrosion and oxidation resistance properties at elevated temperature up to 750 °C [1]. Moreover, the work taken by the authors of this paper proved that K4750 exhibited good weldability due to high liquation cracking and strain age cracking resistance. The investigation on welding of K4750 with other materials is an effective method to comprehensively evaluate its weldability and then extend the use range of the novel designed precipitation hardened superalloy [2]. Hastelloy X is a solid-solution strengthened superalloy with outstanding oxidation and corrosion resistance properties and good strength at elevated temperatures. It is widely used in gas turbine engines for combustion zone components such as transition ducts, combustor cans, spray bars and flame holders as well as in afterburners, tailpipes, and cabin heaters [3,4]. Dissimilar metal joint between the novel designed K4750 alloy and Hastelloy X will certainly be an attractive component due to the excellent comprehensive properties of these two materials. 

Dissimilar-metal structures take advantage of both materials efficiently and result in the possibilities for the flexible design of the component. However, the fusion welding of dissimilar metals will meet many challenges such as the segregation of high- and low-melting phases due to chemical properties mismatch, large residual welding stress related to the physical properties mismatch, and the formation of brittle phases because of metallurgical incompatibility. In this research, both K4750 alloy and Hastelloy X are austenitic nickelbased superalloys, so there are no such huge differences between K4750 and Hastelloy X compared to other mentioned dissimilar metal couples such as Al-Cu and Al-Fe couples. 

In recent years, extensive previous investigations on welding of dissimilar austenitic based alloys have been conducted. Wang et al. [5] investigated the microstructure and interfacial character in dissimilar welding of 316 SS and Inconel 182. The results revealed two alternately distributed typical fusion boundaries: a narrow random boundary and an epitaxial fusion boundary greatly affect joint microhardness. Dissimilar joints between Inconel 718 and AISI 316L SS were performed using Continuous Current Gas Tungsten Arc Welding (CCGTAW) and Pulsed Current Gas Tungsten Arc Welding (PCGTAW) process employing ER2553 and ERNiCu-7 fillers by Ramkumar et al. [6] They reported an unmixed zone was observed at the weld fusion line adjacent to the Inconel 718 for all the weld metals. It was also inferred from the study that PCGTA weld joints employing ERNiCu-7 filler material exhibited better metallurgical and mechanical properties. Sayiram et al. [7] investigated the microstructural character of dissimilar welds between Incoloy 800H and 321 SS using Inconel 82 and Inconel 617 as filler materials. It has been concluded that Inconel 617 filler material is a preferable choice for the joint between Incoloy 800H and 321 SS. Sireesha et al. [8,9] used four types of filler materials corresponding to 316, 16Cr–8Ni–2Mo, Inconel 82, and Inconel 182 to joint 316LN SS and alloy 800. Microstructural features and mechanical properties of the dissimilar joints were investigated. Their results show that the Inconel 82/182 filler materials offer not only adequate solidification cracking resistance but also good mechanical properties. Dupont et al. [10] investigated the effect of filler material compositions on the microstructure and weldability of dissimilar joints between AL-6XN super SS and two nickel-base alloys, Inconel 625, and Inconel 622. Their results reveal that filler materials with small amount of secondary phase and narrow solidification temperature range contribute to superior cracking resistance. Lee et al. [11] investigated the dissimilar joints between nickel-base alloy 690 and SUS304L by shielding metal arc welding with electrodes covered with varies Ti concentration flux coatings. Their results show that the increase of Ti in filler material composition contributes to the columnar dendrite to equiaxial dendrites transformation and thereby, increase the elongation of the welding joints. The filler materials for joining nickel superalloys and 310 SS were widely investigated by previous researchers [12,13,14,15]. They illustrated that nickel base filler materials are the better choice in terms of room temperature mechanical properties. Inconel 617 was selected as the filler material to joint Inconel 617 and 304H SS using GTAW by Pavan et al. [16]. They showed that elements Cr, Mo, and C are mainly distributed in inter-dendritic regions in fusion zone which leads to the formation of M_23_C_6_ type carbides. 

As can be seen from the previously published works, the selection of suitable filler materials is the critical issue in welding of dissimilar Ni-based austenitic alloys. The objective of the present work was to characterize the microstructures and mechanical properties of gas tungsten arc welded dissimilar joints between K4750 alloy and Hastelloy X by varies filler materials, thereby to evaluate the weldability and choose proper filler materials for the new developed cast superalloy K4750.

## 2. Experimental Details 

### 2.1. Materials 

**Figure 1:**
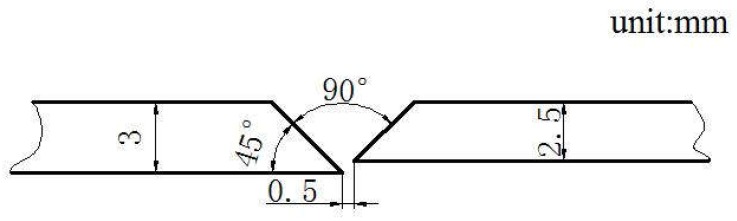
Welding configuration of dissimilar joints.

The materials used were K4750 and Hastelloy X both as plate and filler material. The plate thicknesses used in the study were 3 mm of Hastelloy X alloys and 2.5 mm of K4750 alloy, respectively. The diameter is Ø1.2 mm for both the K4750 and Hastelloy X filler materials. The as-received Hastelloy X and K4750 plates were solutionized at 1120 °C for 4 h followed by air cooling and 1160 °C for 10 min followed by air cooling. Oxidation products at the plate surfaces were removed by using a steel brushing and then a groove was machined at the edge of the plate as shown in Figure 1. The chemical composition of K4750 in weight percent is 0.1C, 19.9Cr, 4.3Fe, 4.5(Mo + W), 4.4(Al + Ti), 1.5Nb, and balance Ni. The chemical composition of Hastelloy X in weight percent is 0.1C, 21.8Cr, 18.5Fe, 9Mo, 0.6W, 1.5Co, and balance Ni. Impurities such as Si, S, and N were ignored in both materials.

### 2.2. Welding Process 

Butt welding of the dissimilar alloys were conducted by manual welding using direct current electrode positive gas tungsten arc welding (GTAW). To avoid oxidation during welding, shielding gas (Ar) with a purity of 99.99% was utilized at both sides of the welding part. An Ø2.0 mm cerium containing tungsten electrode was used. Other detail welding parameters are listed in Table 1. 

### 2.3. Microstructural Evaluation and Defects Test

X-ray non-destructive testing was subjected to the as-welded plates to identify the possible cracks. After that, metallography samples of the dissimilar joints were cut by electro discharge machining and prepared by standard metallographic polishing technique for microstructural study. Thereafter, the specimens were chemically etched by a solution containing 20 g CuSO_4_ + 150 mL HCl + 80 mL H_2_O. The joint microstructures were examined and analyzed by optical microscopy OM (ZIm Carl Zeiss, Oberkochen, Germany), scanning electron microscope SEM (Quanta 600 FEI, Hillsboro, OR, USA), and Electron probe microanalysis EPMA (JXA-8530F, JEOL Ltd., Tokyo, Japan), JEM-2100 transmission electron microscopy TEM (JEM-2100, JEOL Ltd., Tokyo, Japan). The TEM samples were prepared by twin-jet polishing with 10% perchloric acid in 90% methyl alcohol at the temperature of −18 °C and the voltage of 15 V.

### 2.4. Mechanical Properties Test

**Figure 2:**
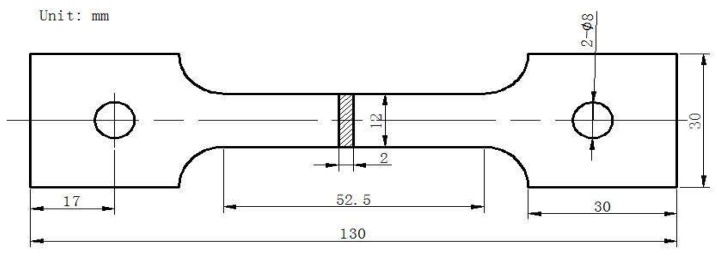
Configuration of tensile test sample.

Vickers micro-hardness test was conducted at the middle line of the metallography samples in the transverse direction with the load of 0.5 kgf and the dwell time of 15 s by a microhardness tester (AMH43, LECO, Sao Jose, MI, USA). The distance between two indentations is 0.5 mm in the fusion zone and base metal, 0.25 mm in the HAZ. The tensile test samples were mechanically machined into the configuration shown in Figure 2. The room temperature tensile tests of the joints under different conditions were performed using tensile test machine (INSTRON5582, INSTRON, Boston, MA, USA). 

## 3. Results 

### 3.1. Microstructure of as-Received Materials

**Figure 3:**
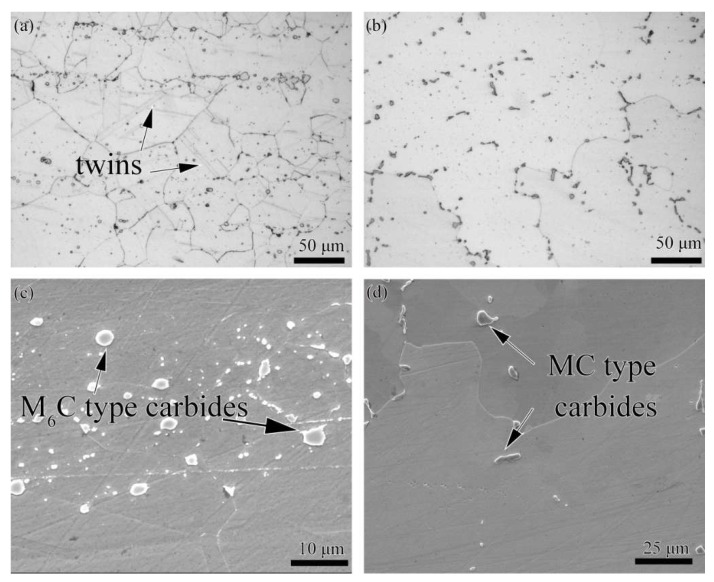
Microstructure of as-received base materials: (**a**) OM image of Hastelloy X alloy; (**b**) OM image of K4750 alloy; (**c**) SEM image of Hastelloy X; and (**d**) SEM image of K4750 alloy.

**Figure 4:**
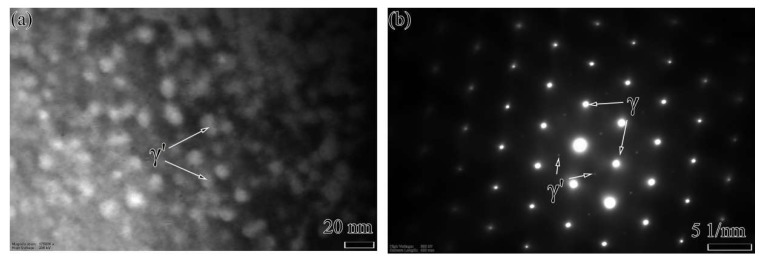
TEM analysis of γ’ in as-received K4750 base metal: (**a**) bright filed image of γ’; and (**b**) SADP (selected area electron diffraction pattern) image of γ’ and γ.

The microstructure of as-received Hastelloy X and K4750 base metal is shown in Figure 3. Figure 3a depicts the Hastelloy X alloy optical microstructure. An austenitic matrix, γ, with equiaxed grains with the size of 80 μm and annealing twins were observed. Striping distributed spherical precipitates can also be distinguished easily. SEM image (Figure 3c) and EDS results (Table 2) of these spherical precipitates show that they are Mo and Cr rich M_6_C type carbides. As for the K4750 alloy, austenitic γ matrix with large grain size and precipitates distributed along interdendrite show the cast feature. The precipitations distributed along interdendrite with the shape of blocky and bar are randomly distributed inside the grains or at the grain boundaries. It is demonstrated by the EDS results shown in Table 2 that these precipitations are Nb and Ti rich MC type carbides. The main strengthen precipitate γ’ is not identified by SEM images due to the rapid cooling rate from the pre-weld solutionized heat treatment. TEM analysis was conducted to examine the detail microstructures. TEM results shown in Figure 4 reveal that γ’ with the size of about 20 nm was formed during cooling from pre-weld solutionized heat treatment. 

### 3.2. Macrostructure of Dissimilar Joints

**Figure 5:**
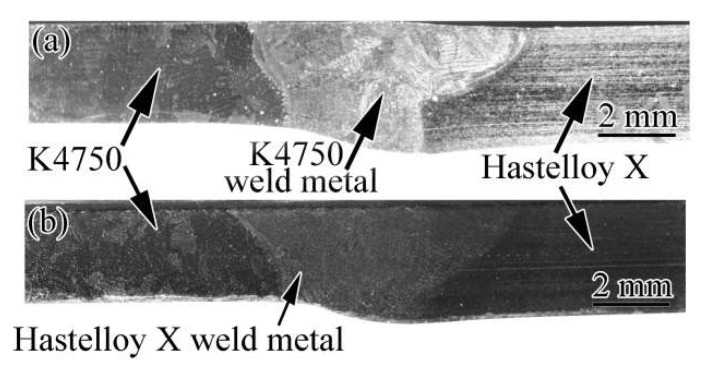
Macrostructure of dissimilar joints with different filler materials: (**a**) K4750 filler material; and (**b**) Hastelloy X filler material.

The cross-section macrographs of the dissimilar joints between K4750 alloy and Hastelloy X in the as-welded conditions with different filler materials are shown in Figure 5. Macrostructure examination ensured that both filler materials employed in this study exhibited proper fusion with the base metals. Further non-destructive testing (NDT) has shown clearly that the dissimilar joints are free from defects including incomplete penetration, porosities, inclusions, etc. 

### 3.3. Microstructure of Fusion Zone 

**Figure 6:**
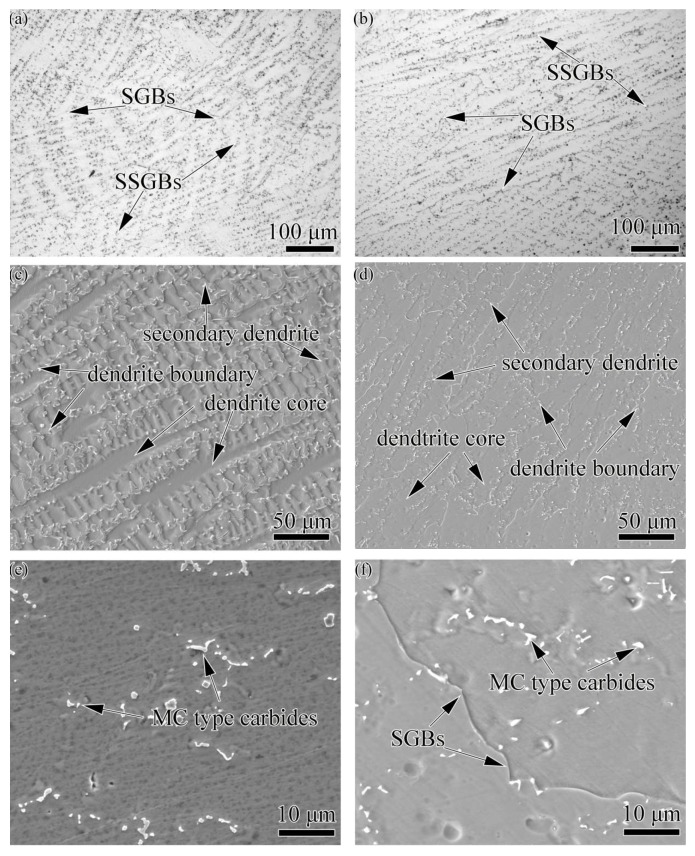
Microstructures of weld metal with Hastelloy X and K4750 filler material: (**a**) OM image of weld metal with Hastelloy X filler material; (**b**) OM image of weld metal with K4750 filler material; (**c**) SEM image of weld metal with Hastelloy X filler material; (**d**) SEM image of weld metal with K4750 filler material; (**e**) precipitates in Hastelloy X weld metal; and (**f**) precipitates in K4750 weld metal.

**Figure 7:**
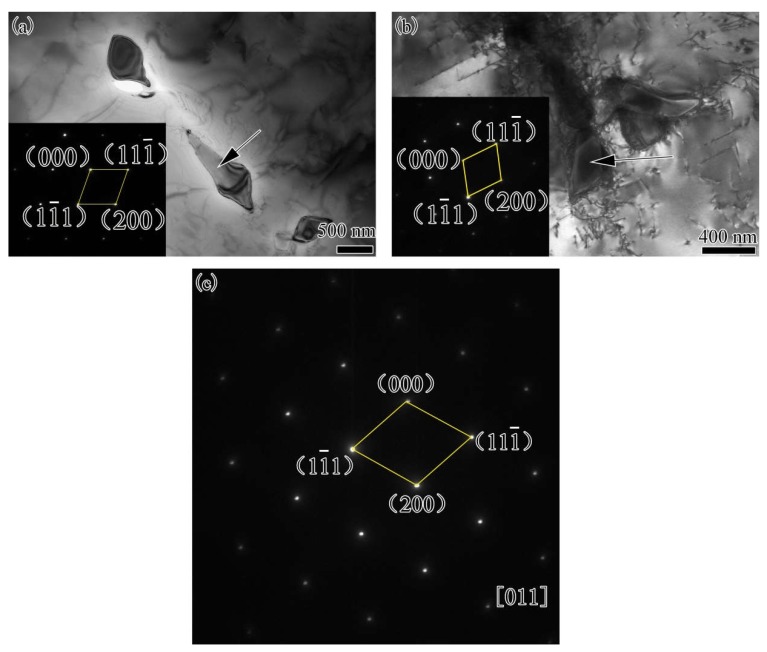
TEM analyses of carbides in weld fusion zone: (**a**) MC carbides in K4750 weld metal and inset is the SADP of the arrow marked MC; (**b**) MC carbides in Hastelloy X weld metal and inset is the SADP of the arrow marked MC; and (**c**) SADP of K4750 weld metal matrix area reveals no superlattice reflections of the γ’ phase.

The microstructure of the weld metals with different filler materials is shown in Figure 6. The typical columnar dendritic microstructure is observed in both dissimilar joint fusion zones. Sub-solidification grain boundaries (SSGBs) and solidification grain boundaries (SGBs) can be observed in both weld metals with different filler materials. Dendritic morphologies with coarsened secondary dendrites can be observed in Hastelloy X weld metal, whereas, in the K4750 weld metal, it is not so obvious. Precipitates which are mainly distributed along the interdendrites are determined to be carbides. It is hard to identify the compositions of these precipitates by EDS analysis due to the small size therefore, TEM was conducted to further examine the precipitates and the results are shown in Figure 7. SADP results of these precipitates proved that precipitates formed in both weld metals are MC type carbides. The TEM/EDS results reveals that these carbides are concentrated in Mo, Ti, and Nb elements but the contents of these elements are different, as shown in Table 3. Carbides formed in the Hastelloy X weld metal exhibits higher concentration of Mo and Nb while that in K4750 weld metal are rich in Nb and Ti. The lattice parameters of these two carbides are 0.4240 and 0.4237 nm, respectively. No γ’ was found in the weld metal and the SADP did not show super-lattice reflections of the γ’ phase. Several areas were detected and a typical example is shown in Figure 7c, where the SADP from the [011] zone axis does not contain γ’ super-lattice reflections.

### 3.4. Elemental Segregation in Different Weld Metals

The elemental concentration of dendrite cores (C_core_) and dendrite boundaries (C_boundary_), as show in Figure 6c,d, were detected by EPMA/WDS. Then, the element partition coefficient k was obtained from the ratio of C_core_ to C_boundary_. The elemental segregation in different filler materials are summarized in Table 4. The segregation degree of most elements in Hastelloy X weld metal is more severe than that in the K4750weld metal. In Hastelloy X weld metal, elements such as Al, Si, Nb, Ti, and Mo are with the values of k smaller than unity, while elements of Fe and W are with the values of k greater than unity. The rest of the elements like Co and Cr are with the values of k very close to unity. However, the segregation in the K4750 weld metal shows some differences. Elements such asSi, Nb, Ti, and Mo are also with the values of k smaller than unity but they are still greater than those in the Hastelloy X weld metal. Iron and W are with the values of k greater than unity and also higher than those in the Hastelloy X weld metal. The k values of elements Co and Al are smaller than unity in the Hastelloy X weld metal but they are greater than unity in the K4750 weld metal. 

### 3.5. Interfacial Characteristics 

**Figure 8:**
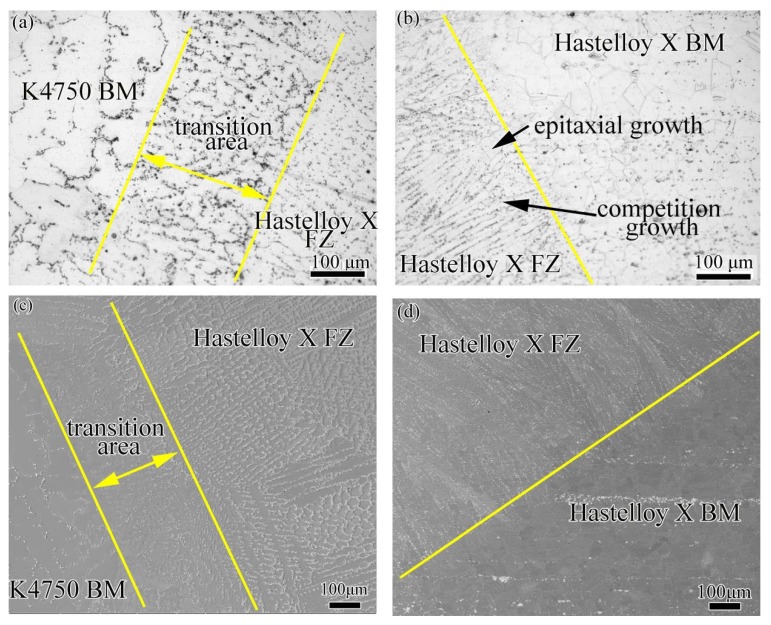
Interfacial characteristics of dissimilar joints with Hastelloy X filler material (**a**) OM image of K4750 (BM) base metal and Hastelloy X (FZ) fusion zone interface; (**b**) OM image of Hastelloy X BM and Hastelloy X FZ interface; (**c**) SEM image of K4750 BM and Hastelloy X FZ interface; and (**d**) SEM image of Hastelloy X BM and Hastelloy X FZ interface.

Figure 8 shows the interfacial characteristics of dissimilar joints with Hastelloy X filler material. The OM and SEM images of interface between Hastelloy X weld metal and K4750 base metal are shown in Figure 8a,c, respectively. A transition area with the maximum width about 200 μm can be clearly detected in OM and SEM images, as shown in Figure 8a,c. Figure 8b,d shows the OM and SEM images of interface between Hastelloy X weld metal and Hastelloy X base metal, respectively. No considerable unmixed zones can be observed in these figures. Epitaxial growth is clearly identified in Figure 8b and the competitive growth in Figure 8d as well. Moreover, the grains growth direction in the weld fusion zone is vertical to the fusion boundary because this is the maximum heat conduction direction. 

**Figure 9:**
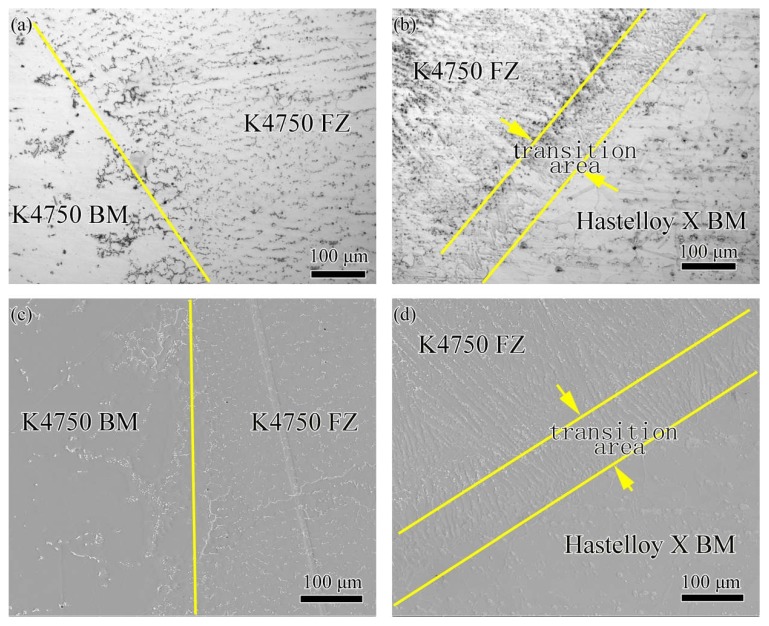
The interfacial characteristics of dissimilar joints with K4750 filler material (**a**) OM image of OM image of K4750 (BM) base metal and K4750 (FZ) fusion zone interface; (**b**) OM image of Hastelloy X BM and K4750 FZ interface; (**c**) SEM image of K4750 BM and K4750 FZ interface; and (**d**) SEM image of Hastelloy X BM and k4750 FZ interface.

Figure 9 shows the interfacial characteristics of dissimilar joints with K4750 filler material. The OM and SEM images of interface between K4750 weld metal and K4750 base metal are shown in Figure 9a,c, respectively. No considerable unmixed zones can be observed in figure. Epitaxial growth of K4750 weld metal from the K4750 base metal can be clearly observed. Figure 9b,d shows the OM and SEM images of interface between K4750 weld metal and Hastelloy X base metal, respectively. A transition area with the maximum width about 100 μm can be clearly detected between K4750 filler material and Hastelloy X base metal from the OM and SEM images.

**Figure 10:**
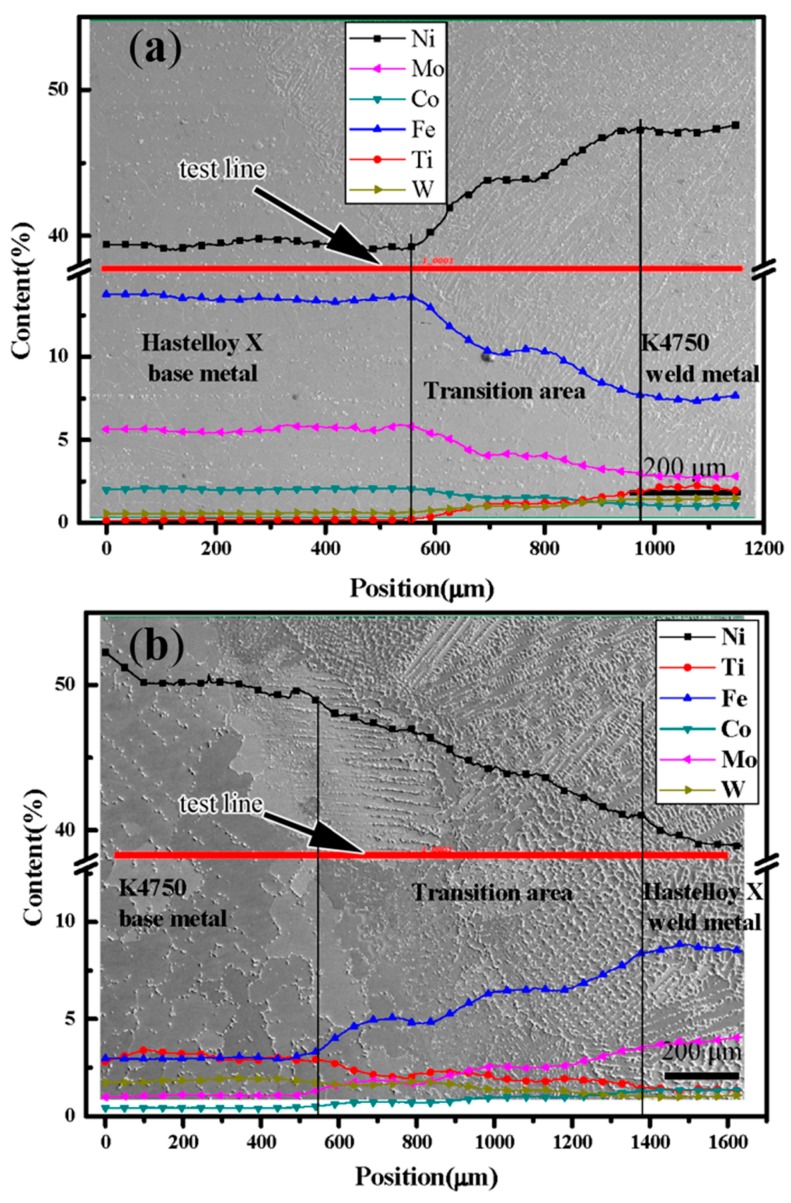
Elements distribution across the interfaces in dissimilar joints (**a**) interfacial element distribution in joints of K4750 filler material; and (**b**) interfacial element distribution in joints of Hastelloy X filler material.

Figure 10 show the elements distribution across the interface of the dissimilar joints with different filler materials by EPMA/WDS. The interface between the Hastelloy X base metal and the K4750 weld metal is shown in Figure 10a and the interface between the K4750 base metal and the Hastelloy X weld metal is shown in Figure 10b. In Figure 10a, a transition area with the width approximately 400 μm can be clearly detected. At the transition area, Ni, W and Ti are gradually increased, whereas Fe, Co and Mo are gradually decreased from the Hastelloy X base metal side to the K4750 weld metal side. Figure 10b shows an elements transition area with the width of approximately 900 μm, which is wider than that in the joint with K4750 filler material. Ni, W and Ti are gradually decreased from the K4750 base metal side to the Hastelloy X weld metal side, whereas Fe, Co and Mo are gradually increased. The unmixed zones are not detected at either interface. The elemental variation trend is mainly related to the chemical compositions of these alloys at both sides of the interface. 

### 3.6. Microhardness Distribution 

**Figure 11:**
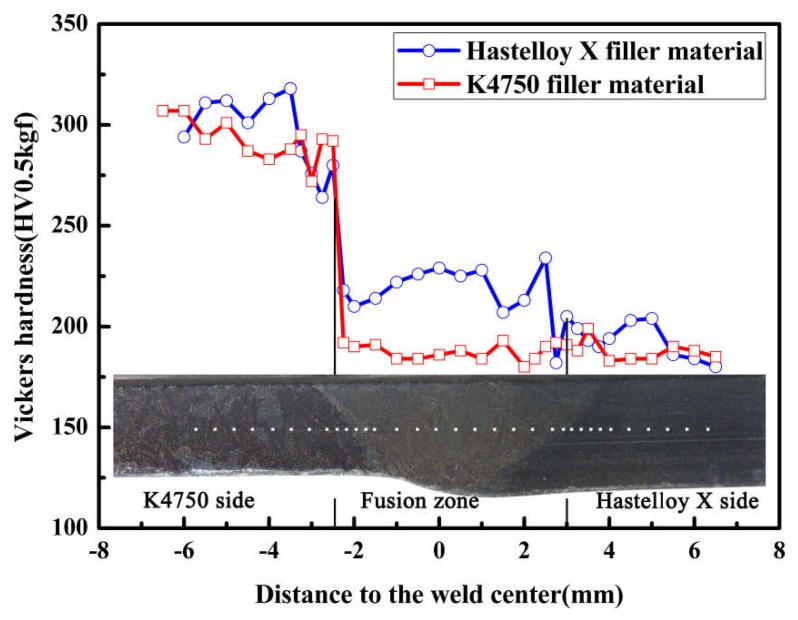
Microhardness distributions of the dissimilar joints with different filler materials.

Hardness studies were carried out across the center line of the joints as shown in the inset of Figure 11. There is a sharp decrement in hardness values from the K4750 superalloy base metal towards the Hastelloy X base metal for both filler materials. The microhardness values of K4750 alloy is approximately 300 HV, while that of the Hastelloy X alloy is approximately 185 HV. The fusion zone hardness values are 185 HV in the Hastelloy X weld metal and 225 HV in the K4750 weld metal, respectively. The hardness in Hastelloy X weld metal is similar to that of Hastelloy X base metal which indicates that the dilution of Hastelloy X weld metal by K4750 base metal does not affect the hardness of Hastelloy X weld metal. 

### 3.7. Tensile Properties of Dissimilar Joints 

**Figure 12:**
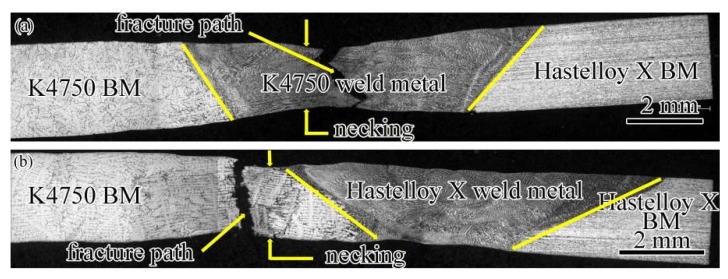
Tensile fractured samples of the dissimilar joints with different filler materials (**a**) K4750 filler material; and (**b**) Hastelloy X filler material.

Table 5 shows the tensile properties of both base metals and dissimilar joints with different filler materials. As can be seen in Table 5, the ultimate tensile strength (UTS) and yield strength (YS) of the dissimilar joints with K4750 filler material are 717.9 and 381.5 MPa, whereas the values are 734.2 and 386 MPa for Hastelloy X filler material. It is worth noting that the mechanical property of the K4750 base metal is tested under the condition of solutionized and aged. The joints with K4750 filler materials are slightly lower than that with Hastelloy X filler materials. However, the total elongation of dissimilar joints with K4750 filler material is higher than that with Hastelloy X filler materials and the values are approximately 28% and 21%, respectively. The fracture location of dissimilar joints with K4750 filler materials is at the weld fusion zone, whereas for the Hastelloy X filler material is at the K4750 HAZ (Figure 12). Significant necking occurred at the weld fusion zone of the joints with K4750 filler material. The joint efficiency (correspond to the Hasetlloy X base metal) of dissimilar joints with K4750 filler material and Hastelloy X filler materials are 98.4% and 101%, respectively. 

### 3.8. Morphology of Fractured Surface

**Figure 13:**
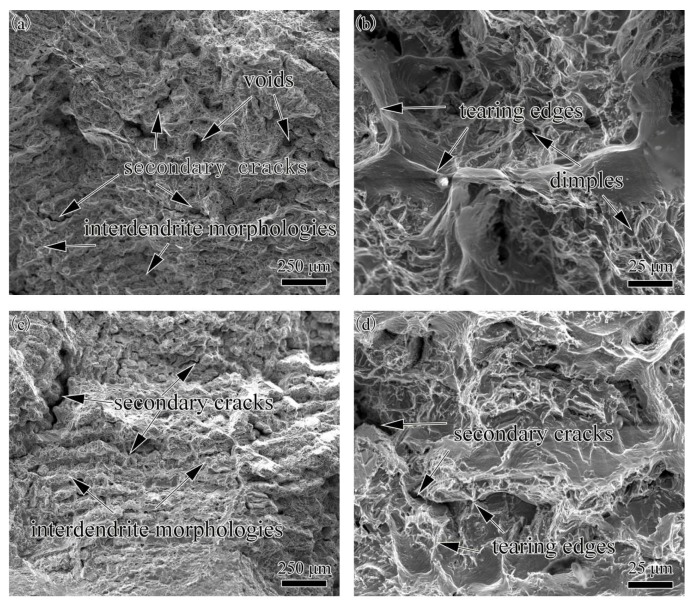
Fractured surface morphologies of dissimilar joints with different filler materials (**a**,**b**) K4750 filler material; and (**c**,**d**) Hastelloy X filler material.

The SEM images of the fractured surfaces of dissimilar joints with different filler materials are shown in Figure 13. Figure 13a,b shows the fractured joints with K4750 filler material. The fractured surfaces of joints with Hastelloy X filler materials are shown in Figure 13c,d. In Figure 13a,c, interdendritic fracture characteristics with secondary cracking and voids can be clearly observed in the fractured surface. The occurrence of tearing edge and dimple-like morphology indicating the fracture mode of both dissimilar joints are ductile fracture. Furthermore, fractured surface of dissimilar joints with K4750 filler material exhibited larger and deeper dimples feature which implies higher ductility during tensile test. This is in good accordance with the results of elongation values in tensile test shown in Table 5.

## 4. Discussions

Microstructural evaluation and mechanical properties assessment of the dissimilar joints between the novel designed K4750 alloy and Hastelloy X have clearly corroborated that both selected filler materials resulted in sound welds which were free from defects and exhibited good mechanical properties. This clearly confirms the excellent weldability between the new designed cast Ni-based K4750 superalloy and Hastelloy X.

### 4.1. Microstructural Evaluation and Phase Transformation

The microstructures of both as-received base metals and as-welded weld metals are shown in Figure 3, Figure 4, Figure 5, Figure 6 and Figure 7. The microstructures of as-received base metals reveal that both materials consisted of austenitic γ matrix and carbides due to the addition of carbon to nickel matrix, as expected. Finer γ’ with the size about 20 nm was also detected in K4750 alloy, as shown in Figure 4. It depicted that the air cooling after pre-weld solutionized heat treatment is unable to completely suppress the formation of γ’ due to high concentration of γ’ former elements, whereas coarsening of γ’ particles was avoided. Pre-weld solution heat treatment resulted in small size γ’ particles which is benefit to heat affected zone (HAZ) cracking resistance properties of superalloys [17,18,19]. Typical weld metal microstructure features with column-dendrites, SGBs and SSGBs can be observed in both weld metals. MGBs, which are usually connected with the formation of solidification cracking and/or ductility-dip cracking, are not detected in both weld metals. The SGBs result from the intersection of packets or groups of subgrains. The formation of SSGBs is related to the alloying elements segregation during solidification. Elements with the partition coefficient value k smaller than unity such as Ti, Nb, and Mo as shown in Table 4 will concentrate in SSGBs. The elemental segregations are further discussed in the following section. The coarsened and developed secondary dendrites in Hastelloy X weld metal is mainly attributed to the slower cooling rate compared to that in the K4750 weld metal, as mentioned in the following section. In addition, the higher amount of Ti and Nb in K4750 weld metal will contribute to the constitutional cooling which leads to the fast cooling rate and further result in less significant secondary dendrite microstructure.

Carbides in K4750 and Hastelloy X base metal are confirmed to be primary MC type [1] and M_6_C type [3] carbides, respectively. In both weld metals, however, these carbides are proved to be MC type carbides (Figure 7). The carbides in K4750 weld metal are MC type because most of the weld metal is K4750 alloy. However, it is surprising that carbides in Hastelloy X weld metal are also MC type, since most of the weld metal consisted of Hastelloy X. The carbides formed in Hastelloy X weld metal is predicted to be M_6_C which is same as the Hastelloy X base metal.

According to the previously reported results, M_6_C is prone to form in an alloy where the amount of Mo + W is greater than 6 wt % [20]. As mentioned above, chemical compositions of Hastelloy X weld metal are a mixture of K4750 and Hastelloy X alloys. The maximum amount of Mo + W in Hastelloy X weld metal is about 6.6 wt % which is greater than 6 wt % (Table 4). The contradictory result shows it is controversial to use this criterion to predict the formation of M_6_C type carbides in superalloys. It should be noted that M_6_C type carbide has a variable composition, and the actual concentration of elements may depend on the alloying systems. The absence of M_6_C carbides in an alloy contained Mo + W concentration over 6 wt % is also reported by researchers. For example, Collins et al. [21] reported that no M_6_C type carbides were observed in an alloy contained Mo + W concentration of 7.4 wt %. In contrast, M_6_C type carbide was found in other superalloys that contained Mo + W < 6 wt % [22]. Thus, it is possible that some other factors in addition to the concentration of Mo + W control the formation of the M_6_C type carbide precipitates in the Hastelloy X weld metal. The segregation of minor elements especially boron may be another factor. IDOWU et al. [23] reported that the segregation of boron from the interlayer towards the adjacent base metal contributes to the precipitation of M_6_C type carbide in the base metal. However, no significant segregation of boron was observed in Hastelloy X weld metal. Therefore, M_6_C type carbides were not formed in Hastelloy X weld metals though the amount of Mo + W is greater than 6 wt %.

The MC type carbide is a high-temperature precipitated carbide formed at the final stage of weld metal solidification [24]. The formation of MC type carbide results in the decrement of carbon concentration in the weld metal which also reduces the formation tendency of low temperature carbides such as M_6_C. MC carbides formed in Hastelloy X weld metal exhibit larger lattice parameters as compared to those in K4750 weld metal which is attributed to the reduction of larger-diameter atomic elements such as Mo and Nb (Table 3). No γ’ was identified in both weld metals due to decrement of Al and Ti concentrations resulting from the dilution of K4750 by Hastelloy X, combined with the rapid cooling rate in the weld metal.

### 4.2. Segregation Behavior

Elemental segregations in different filler materials are shown in Table 4. As can be seen from Table 4, most of the alloying elements show same segregation tendency in different weld metals. More specifically, Si, Nb, Ti, and Mo with the k values smaller than unity tended to segregate to interdendritic regions, while Fe and W with the k values greater than unity are prone to distribute in dendrite cores. Chromium shows no segregation tendency in both weld metals with the k value very close to unity. The result is in well agreements with those reported in other superalloy weld metals [25,26,27]. It is notable to see that interdendritic region segregation alloying elements such as Nb, Ti, and Mo are also strong carbide formers. This phenomenon can be used to explain the carbides distribution along interdendritic regions.

Generally, elements in Hastelloy X weld metal shows more server segregation degree than that in K4750 weld metal. As is well known, element segregation is a time dependent procedures. When the weld metal solidified with a slow rate, there is sufficient time for solute elements re-distribution, thus the elements microsegregation degree is severe. The melting temperature range of Hastelloy X is from 1260 °C to 1355 °C [28], whereas the K4750 alloy is between 1270 °C and 1350 °C [29]. Wider melting temperature range of Hastelloy X filler material leads to slower cooling rate and longer solidification time within the fusion zone as compared to the K4750 filler material. This result was also proved in GTAW [30] and EBW (Electron Beam Welding) [31] produced weld fusion zones. They showed that element segregation degree in GTAW fusion zone is more severe than that in the EBW fusion zone due to the slower cooling rate of GTAW fusion zone.

On the other hand, there are also some elements like Co and Al showing opposite segregation behavior in different weld metals. The opposite segregation behavior of Co could be attributed to the solute interactions. Dupont et al. [32] proved that the increment of Fe content will result in the decrement of k value of Nb. The same phenomenon was also observed by Dupont for Mo [33]. They explained that the addition of Fe can decrease the solubility of Nb and Mo in nickel matrix due to the smaller ultimate solubility of Nb in Fe-Nb than that in Ni-Nb and Mo in Ni-Mo than that in Fe-Mo according to the binary phase diagram systems. Accordingly, the ultimate solubility of Al and Co in nickel is much bigger than that in iron as shown in the phase diagram [34]. Obviously, as shown in Table 4, the content of Fe in Hastelloy X weld metal is higher than that in K4750 weld metal. Thus, as the dendrites formed with higher Fe concentration in Hastelloy X weld metal, the decreased solubility will lower the amount of Al and Co dissolved in the dendrite cores and increased segregation to dendrite boundaries will occur. Therefore, the k values of Al and Co will be decreased in Hastelloy X weld metal as compared to K4750 weld metal. On the other hand, the segregation behavior of Al and Co certainly will influence the behavior of other elements, such as W. Furthermore, it is well known that during dendritic solidification, the segregation degree can be significantly reduced by solid state diffusion, which would be more pronounced with slowly cooled Hastelloy X weld metal.

### 4.3. Interfacial Microstructures

Figure 8, Figure 9 and Figure 10 show the interfacial characteristics of dissimilar joints with different filler materials. Transition areas can be found between K4750 base metal and Hatelloy X weld metal, Hastelloy X base metal and K4750 weld metal interfaces, respectively. However, the former one is larger than that of the later one. The formation of transition area is commonly in dissimilar metal joints due to the chemical and metallurgical properties difference of both materials. Unmixed zones which, are also frequently reported in the dissimilar materials joint, did not occur in the present study. The occurrence of transition areas between the Hastelloy X alloy and the K4750 alloy is mainly attributed to their chemical compositions difference, as mentioned in the Section 2.1. Such a gradual change of these elements is possibly related to the dilution and element diffusion during welding. The K4750 alloy presents higher content of Ti and W whereas lower content of Fe, and Mo compared to Hastelloy X alloy. Locally melted Hastelloy X base metal induced by welding arc was diluted by the K4750 filler material molten drops which result in the decrease of Fe, Mo, and Co content and the increase of Ti and Ni. The rapid solidification weld metal couldn’t supply enough time for element diffusion which restricted the completely homogenization of the element across the interface. Thereafter, the transition area located at the joint interface is formed. The larger transition area can be explained by the wider melting temperature range of Hastelloy X filler material than that of the K4750 filler material. Wider melting temperature range of Hastelloy X filler material leads to slower cooling rate and longer solidification time as compared to the K4750 filler material. When the weld metal solidified with a slow rate, there is sufficient time for solute elements diffusion, although the diffusion is not enough to completely homogenize the interfacial chemical compositions, thus the element transition range at the interface is wider.

When the melting range of filler materials approaches or exceeds the melting range of the base metal, only a small fraction of the base metal can be melted and without dilution in the re-solidification stage; therefore, no unmixed zone is formed between two regions [35,36]. Kourdani [37] reported that no obvious unmixed zone will form when the melted base metal dilution by filler material occurs in the re-solidification stage. Both K4750 filler material and Hastelloy X base metal are Ni-base and exhibit FCC (Face Center Cubic) structure, mixture of these two alloys can easily take place under the effect of weld arc force and surface tension force. The mixture of these two melted alloys will certainly result in the dilution of the welded alloy at the weld interface which indicating the absence of unmixed zone is reasonable and further proving good compatibility of these two superalloys in fusion welding process. Unmixed zone is reported to be detrimental to the corrosion resistance of dissimilar weld joints and narrower or free of unmixed zone dissimilar joint is able to obtain by using ultrasonic vibration assistance [35].

### 4.4. Mechanical Properties

Figure 11 depicts the microhardness profile of dissimilar joints with varies filler materials. Microhardness measurements showed that the average hardness of K4750 weld metal was greater than that of Hastelloy X weld metal. The higher hardness at K4750 weld metal is mainly attributed to the higher content of Ti and Al elements as compared to the Hastelloy X weld metal. Although no γ’ was observed in the K4750 weld metal, the relatively high concentration of alloying elements such as Al and Ti certainly will improve the microhardness of the area. However, it is unable to recover the microhardness to the level of K4750 base metal due to the element dilution effect and faster cooling rate from weld metal solidification. It is noted to observe the equal hardness values between the Hastelloy X weld metal and Hastelloy X base metal. This indicates that small amount of Al, Ti, and Nb from K4750 base metal in the Hastelloy X weld metal does not contribute to the hardness increment of the area. This phenomenon can also be explained that the hardness increment result from the increment of Al, Ti, and Nb is equal to the hardness decrement result from the reduction of Mo and Co elements concentration. Moreover, the finer dendrities in the K4750 weld metal as shown in Figure 6d is also responsible for the higher hardness as compared to the Hastelloy X weld metal.

The tensile tests results of both base metals and dissimilar joints are shown in Table 5. Sound dissimilar joints with the joint efficiency of 98.4% and 101% were obtained by K4750 filler material and Hastelloy X filler material, respectively. Hereby, we suggest the use of Hastelloy X filler materials to joint these two alloys for superior comprehensive mechanical properties. The Hastelloy X base metal exhibit excellent ductility due to the solid solution strengthen nature, which further results in the good ductility of both dissimilar joints. Relatively higher total elongations of the joints with K4750 filler material than that with Hastelloy X filler material is mainly attributed to the finer dendritic microstructures. Lee et al. [10] observed the same results that the addition of Ti elements in filler material results in finer dendritic microstructure due to the occurrence of high constitutional cooling, thereby significantly promoting the elongation of the dissimilar joint. Fracture surface morphologies of both dissimilar joints presents ductility fracture mode with tearing edges and dimple-like features. Fracture locations of both dissimilar joints reveal that Hastelloy X filler materials contributes to higher room temperature UTS and YS than those of the K4750 filler material and K4750 base metal.

The presented results are major contributions to the existing literature and also open a wide range of research opportunities in the bimetallic joints employing new developed K4750 superalloy and Hastelloy X. However, as a new precipitation hardened superalloy aimed to use at temperatures around 750 °C, high temperature mechanical properties of the dissimilar joints and post weld heat treatment regimens need to be considered, therefore the high temperature tensile test of the dissimilar joints and suitable PWHT (Post Weld Heat treatment) process will be studied in the future.

## 5. Conclusions

Dissimilar metals between a new cast superalloy K4750 and Hastelloy X were butt welded by gas tungsten arc welding process. Two types of filler materials with same chemical compositions as K4750 and Hastelloy X were used in the present work.

The following conclusions can be drawn from the results:

1. Sound dissimilar joints between a new cast superalloy K4750 and Hastelloy X could be obtained by GTAW process using K4750 and Hastelloy X filler materials.
2. Carbides in both weld metals are proved to be MC type rather than M_6_C type. No γ’ was observed in either weld metal.
3. The segregation degree of various elements in Hastelloy X weld metal is severe than that in the K4750 weld metal. Opposite segregation behavior of elements Al is mainly attributed to the higher amount of Fe element.
4. No unmixed zones are observed at the interfaces. Transition areas with the chemical compositions various between the K4750 alloy and the Hastelloy X alloy are found at the K4750 alloy and Hastelloy X alloy interfaces. The maximum width of the transition area between the K4750 weld metal and Hastelloy X base metal is 400 μm which is smaller than that between the Hastelloy X weld metal and K4750 base metal with the width of 900 μm.
5. The dissimilar joints with K4750 filler material exhibit higher microhardness than that with Hastelloy X filler material. Both joints were fractured with a ductile mode and the joint efficiency of dissimilar joints with K4750 filler material and Hastelloy X filler material are 98.4% and 101%, respectively.

## Figures and Tables

**Table 1:** Specific welding parameters in this study.

Welding Current (A)	Welding Voltage (V)	Nozzle Diameter (mm)	Argon Flow Rate (L/min)
45	10–12	Ø8–16	8–10

**Table 2:** Compositions of carbides in the base metals (Atomic %).

	C	Ti	Nb	Cr	W	Fe	Mo	Ni
M6C	27.85	-	-	20.41	1.58	8.29	20.82	21.05
MC	43.32	18.48	14.44	6.15	2.67	1.05	-	13.89

**Table 3:** MC carbide compositions in fusion zone with various filler material (Atomic %).

Filler Material	C	Cr	Fe	Nb	Ti	W	Mo	Ni
Hastelloy X	44.2	4.6	0.4	13.8	16.5	1.5	17.8	1.2
K4750	61.0	2.3	0	12.5	16.6	1.7	5.7	0.3

**Table 4:** Elements segregation coefficients of weld fusion zone with varies filler materials (wt %).

Filler Material	Locations	Co	Fe	Cr	Al	Si	W	Nb	Ti	Mo
Hastelloy X	C_core_	1.04	13.4	20.54	0.38	0.04	0.75	0.18	0.67	4.80
	C_boundary_	1.06	12.63	21.34	0.43	0.10	0.72	0.33	1.12	5.85
	*k*	0.98	1.06	0.96	0.88	0.4	1.04	0.55	0.59	0.82
K4750	C_core_	0.84	11.04	21.07	0.59	0.10	1.23	0.37	1.00	3.68
	C_boundary_	0.73	10.19	20.79	0.53	0.12	1.08	0.62	1.54	4
	*k*	1.15	1.08	1.01	1.11	0.83	1.14	0.60	0.65	0.92

**Table 5:** Tensile properties of base metals and dissimilar joints with varies filler materials.

Sample	UTS/MPa	YS/MPa	Elongation/%	Fracture Location
K4750BM	956 ± 26	690.5 ± 3.5	5.1 ± 0.8	/
Hastelloy X BM	730	350	53	/
K4750 joint	717.9 ± 12.4	381.5 ± 9.5	27.7 ± 3.3	Fusion zone
Hastelloy X joint	734.2 ± 18.6	386 ± 5	21.1 ± 0.5	K4750 HAZ
